# Radiofrequency, Cryoablation, and Pulsed Field Ablation for Atrial Fibrillation: Mechanisms, Preclinical Evidence, and Clinical Outcomes

**DOI:** 10.3390/biomedicines14040825

**Published:** 2026-04-04

**Authors:** Andrei Mihordea, Mihai Puiu, Gelu Simu, Ioan-Alexandru Minciuna, Radu Rosu, Gabriel Gusetu, Dana Pop, Gabriel Cismaru

**Affiliations:** 1 Cardiology Department, Rehabilitation Hospital, 400066 Cluj-Napoca, Romania; andrei.mihordea@elearn.umfcluj.ro (A.M.);; 2 Fourth Department of Internal Medicine, Cardiology-Rehabilitation, “Iuliu Hatieganu” University of Medicine and Pharmacy, 400347 Cluj-Napoca, Romania

**Keywords:** pulsed field ablation, radiofrequency, cryoablation, catheter ablation, atrial fibrillation, complications, safety, efficacy

## Abstract

Catheter ablation has become a cornerstone therapy for atrial fibrillation, with pulmonary vein isolation as its mechanistic foundation. Radiofrequency ablation and cryoablation, the two established thermal technologies, have demonstrated robust efficacy across multiple randomized trials but remain limited by collateral tissue injury inherent to heat- or cold-mediated lesion formation. Pulsed field ablation has recently emerged as a novel non-thermal energy source based on irreversible electroporation, offering myocardial-selective injury with relative sparing of adjacent structures. This review synthesizes evidence across three complementary domains: fundamental studies; preclinical evidence; and clinical data supporting radiofrequency ablation, cryoablation, and pulsed field ablation for atrial fibrillation. We summarize mechanistic differences in lesion formation, key animal studies that established safety and efficacy profiles, and pivotal randomized clinical trials, including recent head-to-head comparisons and meta-analyses of randomized controlled trials. By synthesizing these levels of evidence, the review aims to place recent clinical results into a mechanistic and translational context. Available evidence demonstrates that pulsed field ablation achieves rhythm-control efficacy comparable to radiofrequency and cryoablation while offering procedural efficiency and a potentially improved safety profile. However, long-term durability data and broader experience remain limited. Understanding the strengths and limitations of each ablation modality is essential for informed clinical decision-making as non-thermal ablation technologies enter routine practice.

## 1. Introduction

Atrial fibrillation (AF) is the most common sustained cardiac arrhythmia and is associated with increased risks of stroke, heart failure, mortality, and impaired quality of life [1,2]. Over the past two decades, catheter ablation has evolved from a second-line therapy to an established rhythm-control strategy for symptomatic AF, supported by multiple randomized trials demonstrating superiority over antiarrhythmic drug therapy and, in selected populations, prognostic benefit [3,4,5]. Central to modern ablation strategies is durable pulmonary vein isolation (PVI), reflecting the seminal observation that ectopic activity originating from the pulmonary veins plays a critical role in AF initiation and maintenance [6].

Radiofrequency ablation (RFA) was the first widely adopted catheter-based energy source for AF ablation and remains the most extensively studied modality. Its efficacy is supported by decades of experimental and clinical investigation, including randomized trials demonstrating improved arrhythmia-free survival compared with pharmacological therapy [3,7]. Mechanistically, RFA relies on resistive and conductive heating to produce coagulative myocardial necrosis, resulting in effective lesion formation but also non-selective thermal injury to adjacent tissues [8,9,10]. Despite advances such as irrigated catheters, contact-force sensing, and lesion quality indices, the risk of collateral injury—including esophageal damage, pulmonary vein stenosis, and phrenic nerve injury—has not been completely eliminated [11].

Cryoablation emerged as an alternative thermal modality, offering lesion formation through freezing-induced cellular injury and a distinct biophysical profile compared with RFA [12,13]. The development of cryoballoon ablation (CBA) technology enabled a single-shot approach to PVI, improving procedural reproducibility and reducing operator dependence. Randomized clinical trials have demonstrated that CBA is non-inferior to RFA for paroxysmal AF, with comparable efficacy and safety outcomes [14]. Nevertheless, cryoablation remains a non-selective thermal technique and is associated with characteristic complications such as phrenic nerve palsy, reflecting its mechanism of action [15,16,17].

Pulsed field ablation (PFA) represents a paradigm shift in AF ablation by employing irreversible electroporation to induce myocardial cell death through electrical membrane disruption rather than thermal injury [18,19,20]. Preclinical studies consistently demonstrate that cardiomyocytes exhibit lower electroporation thresholds than surrounding tissues, resulting in myocardial-predominant lesion formation with preservation of extracellular matrix, vasculature, and adjacent non-cardiac structures [21,22,23,24]. Early human feasibility studies confirmed the ability of PFA to achieve rapid and durable PVI with minimal collateral injury [25], and subsequent randomized trials have demonstrated efficacy comparable to established thermal ablation technologies [26]. This growing interest in non-thermal technologies is driven by the limitations of thermal ablation, including non-selective tissue injury and collateral damage.

Most recently, large randomized controlled trials and meta-analyses have directly compared PFA with RFA and CBA, demonstrating non-inferiority for arrhythmia-free survival and similar overall safety profiles, with consistent signals of reduced collateral tissue injury and improved procedural efficiency [27,28,29]. In this evolving landscape, an integrated overview of the mechanistic foundations, experimental evidence, and clinical trial data supporting RFA, cryoablation, and PFA is essential to contextualize the role of each ablation modality in contemporary AF management. The aim of this review is to provide a structured, mechanistic-to-clinical comparison of RFA, CBA, and PFA to better inform clinical decision-making.

## 2. Biophysical Mechanisms of Lesion Formation

In AF ablation, RFA, CBA, and PFA differ fundamentally in how energy is delivered to tissue and how cardiomyocytes are injured and ultimately eliminated, and these mechanistic differences explain much of their distinct safety and lesion characteristics.

**Figure 1:**
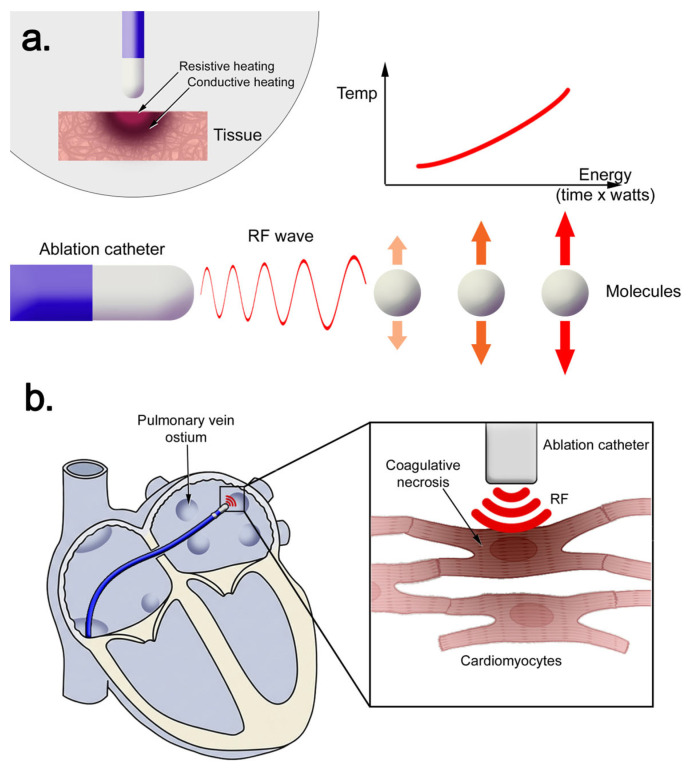
Biophysical mechanism of lesion formation in radiofrequency ablation: (**a**) Physical basis of tissue heating through radiofrequency application. (**b**) Radiofrequency induces cell death through coagulative necrosis. Abbreviations: RF—radiofrequency.

**Radiofrequency ablation** relies on alternating electrical current in the kilohertz range delivered through a catheter tip in contact with myocardium, with tissue injury mediated by resistive and conductive heating rather than direct electrical toxicity [8,9,10]. As tissue temperature exceeds approximately 50 °C [9], protein denaturation, lipid membrane disruption, mitochondrial failure, and microvascular injury occur, producing coagulative necrosis (Figure 1) and initiating an inflammatory–fibrotic healing response [8,10]. Because thermal energy propagates along temperature gradients, lesion formation is non-selective, and collateral injury to adjacent structures may occur [9,10].

**Figure 2:**
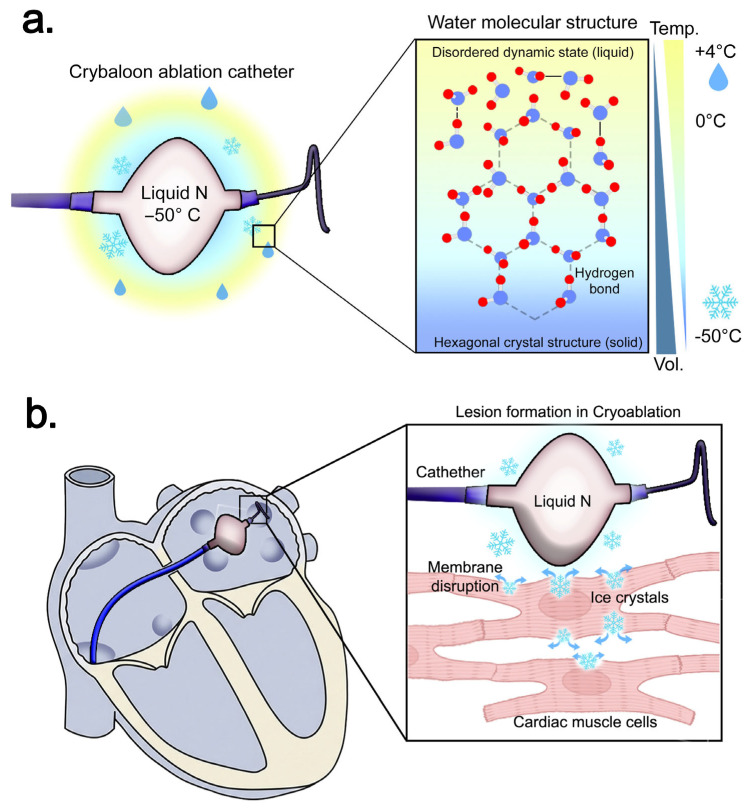
Biophysical mechanism of lesion formation in radiofrequency ablation. (**a**) Physical basis of ice crystal formation and increase in water volume at low temperature. (**b**) Cryoablation-induced injury through cell membrane mechanical disruption by ice crystal formation. Abbreviations: N—nitrogen, Temp.—temperature, and Vol.—volume.

**Cryoablation** induces myocardial injury through rapid heat extraction, typically via nitrous oxide expansion, leading to tissue temperatures below −40 °C [12,13]. During freezing, intracellular and extracellular ice crystal formation causes mechanical disruption of cellular membranes and organelles (Figure 2), while the thawing phase produces cellular volume expansion through osmotic stress and subsequent microvascular stasis, resulting in delayed necrosis and apoptosis [12,13]. Preservation of the extracellular matrix and endothelial integrity leads to more homogeneous lesions and reduced thrombogenicity compared with RFA [30]. Importantly, the freezing phase causes intracellular ice formation (Figure 2), whereas the thawing phase contributes to osmotic stress and microvascular injury.

**Figure 3:**
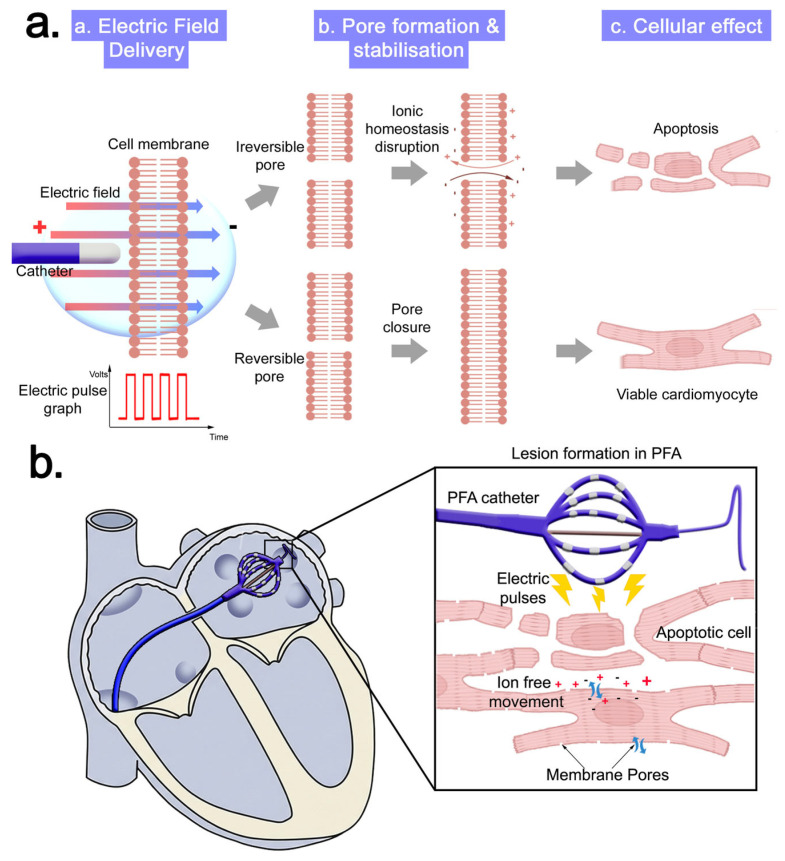
Biophysical mechanism of lesion formation in pulsed field ablation: (**a**) Electroporation of the cardiac muscle cell membrane. (**b**) Pulsed field ablation induced injury through irreversible electroporation, ionic homeostasis disruption and subsequent cell death. Abbreviations: PFA—pulsed field ablation.

**Pulsed field ablation** produces tissue injury via irreversible electroporation, in which high-voltage, ultra-short electrical pulses generate nanoscale membrane pores that disrupt ionic homeostasis and transmembrane potentials [18,19,20]. When the electric field strength exceeds a critical threshold, membrane defects fail to reseal, leading to rapid activation of cell death pathways (Figure 3) with minimal thermal contribution [20]. Cardiomyocytes exhibit increased susceptibility to electroporation due to their geometry and electrical properties, conferring relative tissue selectivity and sparing of non-cardiac structures such as the esophagus and vasculature [21,22,23]. Histopathologic studies demonstrate preservation of collagen, vascular scaffolding, and extracellular matrix architecture following PFA [20,21]. This mechanism enables myocardial-selective ablation with relative sparing of adjacent structures such as the esophagus and phrenic nerve.

**Figure 4:**
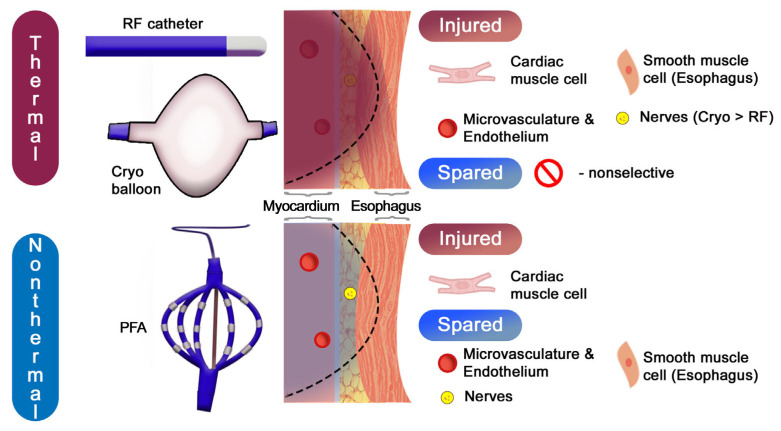
Collateral injury risk and tissue selectivity across different ablation techniques. Abbreviations: Cryo—cryoablation, PFA—pulsed field ablation, and RF—radiofrequency.

In summary, radiofrequency and cryoablation both achieve pulmonary vein isolation through thermal injury—one via heating and the other via freezing—resulting in non-selective cell death and lesion formation that depends on energy transfer and tissue perfusion (Figure 4). PFA, by contrast, eliminates atrial myocardium through electrical disruption of cell membranes, producing rapid, myocardial-predominant injury with minimal collateral damage (Table 1). These biophysical and cellular distinctions underpin the evolving clinical role of PFA as a potentially safer and more tissue-specific modality for atrial fibrillation ablation.

## 3. Preclinical Evidence from Animal Models

### 3.1. Radiofrequency: Animal Studies

Animal studies have played a central role in establishing the fundamental physics and biological effects of radiofrequency (RF) energy used for AF ablation. Long before widespread clinical pulmonary vein isolation, experimental work in canine, porcine, and ovine models clarified that RFA is a thermal injury process, rather than a direct electrical phenomenon [8,31,32]. Seminal investigations led by David Haines, who was a pioneer in the development of RFA, demonstrated that RF lesions are created through resistive heating at the electrode–tissue interface, followed by conductive heat transfer into deeper myocardial layers [8,10]. These studies established the relationships between power, duration, electrode characteristics, and lesion geometry, and identified critical safety thresholds such as impedance rise and steam pops when tissue temperatures approach boiling [10,33].

Subsequent animal experiments refined our understanding of catheter–tissue interaction. Insufficient catheter contact force produces shallow, non-transmural lesions despite adequate power delivery [10], whereas excessive force markedly increases the risk of perforation and steam pops [34,35]. These findings provided the experimental basis for the development of contact-force sensing catheters and force-guided ablation strategies [36]. Parallel studies using canine and ovine models compared irrigated and non-irrigated RF catheters demonstrated that saline irrigation allows higher power delivery with less surface overheating, resulting in deeper and more homogeneous lesions while reducing char and thrombus formation [37,38,39].

Animal models were also instrumental in defining AF-specific ablation strategies. Experimental atrial preparations demonstrated that continuous circumferential lesions are required for durable pulmonary vein isolation and that even small gaps permit electrical reconnection [40,41]. Importantly, animal atrial models showed that conductive thermal spread beyond the atrial wall can injure adjacent structures such as the esophagus, providing a mechanistic explanation for atrio-esophageal injury observed clinically and informing modern posterior wall power and duration limits [42]. More recent porcine studies of high-power, short-duration RFA demonstrated that altering energy delivery shifts lesion geometry toward wider but more superficial injury, with potential implications for both safety and durability [43]. Collectively, these animal studies form the mechanistic foundation of modern RFA and remain the benchmark against which newer non-thermal technologies are evaluated.

### 3.2. Cryoablation: Animal Studies

Animal studies have been fundamental in defining the biophysical mechanisms and lesion characteristics of cryothermal ablation for AF. In contrast to RF energy, cryoablation produces tissue injury through freezing-induced cellular damage, microvascular disruption, and delayed apoptosis rather than immediate thermal coagulation [12,13]. Early canine and porcine experiments demonstrated that cryothermal lesions are created by rapid cooling at the catheter–tissue interface, followed by intracellular ice crystal formation, membrane rupture, and subsequent inflammatory and fibrotic remodeling during the thawing phase [12,13]. These studies also established that lesion depth and durability depend on minimum temperature achieved, duration of freezing, and rate of thaw, rather than power delivery.

Foundational research on animal models showed that cryoablation preserves extracellular matrix architecture, resulting in sharply demarcated lesions with less disruption of endocardial surface integrity compared with RFA [30]. This structural preservation explained the reduced thrombogenicity observed in early cryoablation models and supported its use in left atrial procedures [30]. Importantly, cryothermal energy was shown to maintain catheter adherence to tissue during freezing, improving stability and lesion continuity in beating-heart canine models [44].

Animal models were also critical in the development of CBA for PVI. Canine atrial studies demonstrated that circumferential cryothermal applications at the pulmonary vein ostia could achieve durable electrical isolation, provided that complete balloon–tissue contact and sufficiently low nadir temperatures were achieved [45,46,47]. These experiments identified pulmonary vein size, balloon occlusion, and freeze duration as key determinants of transmurality. Incomplete contact or higher nadir temperatures resulted in reversible injury and late reconnection, highlighting the importance of lesion geometry in cryoablation efficacy [47].

Safety-focused animal studies further clarified the tissue selectivity of cryothermal energy. Porcine models demonstrated relative resistance of collagen-rich structures such as the esophagus and pulmonary vein wall to cryothermal injury compared with RF [48], while also identifying phrenic nerve vulnerability due to cold-induced axonal injury [17]. These findings directly informed clinical strategies such as phrenic nerve pacing during right sided cryoablation. Collectively, animal studies established the mechanistic foundation of cryoablation, defined its unique lesion biology, and provided the experimental basis for its widespread clinical adoption in AF ablation.

### 3.3. Pulsed Field Ablation: Animal Studies

Animal studies have been central to defining the biophysical mechanisms and tissue selectivity of PFA for AF. Unlike thermal energy sources, PFA induces myocardial injury through irreversible electroporation, in which high-intensity, short-duration electric fields create permanent nanopores in cell membranes, leading to loss of cellular homeostasis and delayed cell death rather than coagulative necrosis [20,49]. Early animal experiments demonstrated that myocardial tissue has a lower electroporation threshold than surrounding structures, such as the esophagus [50], phrenic nerve [51], and coronary arteries [21], providing the mechanistic basis for the tissue selectivity observed with PFA.

Experimental animal studies established that PFA lesions are non-thermal, with minimal temperature rise and preservation of extracellular matrix architecture [52,53,54]. Histological analyses in porcine ablated myocardium showed sharply demarcated zones of myocyte death with intact collagen, vasculature, and endocardial surfaces [49,53], contrasting with the coagulative necrosis and thrombogenic surfaces typical of RF lesions. Lesion size and transmurality were shown to depend primarily on electric field strength, pulse number, and waveform, rather than contact force or convective cooling, resulting in highly reproducible lesion geometry across varying atrial wall thicknesses [23,49].

Animal AF models were also critical in validating PFA for PVI. Porcine studies demonstrated that circumferential PFA applications around the pulmonary vein antra reliably achieved acute and durable electrical isolation, with low rates of reconnection on chronic follow-up [23,54,55]. Importantly, adjacent structures exposed to the electric field—most notably the esophagus—showed minimal or no histological injury, even when directly in contact to the posterior left atrial wall [55]. Additional safety studies confirmed relative sparing of the phrenic nerve [23,51] and coronary arteries [21], despite transient stunning at higher field strengths [56], highlighting the steep dose–response relationship characteristic of electroporation.

Collectively, animal studies established that PFA produces cardiac-selective, non-thermal lesions with preserved tissue scaffolding and reduced collateral injury, providing the mechanistic rationale for its favorable safety profile observed in early human trials. This pre-clinical body of work distinguishes PFA fundamentally from both RFA and CBA and underpins its emergence as a novel energy source for AF ablation. However, data from animal studies should be interpreted with caution, as preclinical models are typically characterized by short follow-up periods and tightly controlled experimental conditions, and their results are not always directly translatable to humans, thereby necessitating validation in clinical studies.

## 4. Clinical Outcomes of Catheter Ablation for Atrial Fibrillation

### 4.1. Radiofrequency: Clinical Studies

Randomized clinical trials have firmly established RF catheter ablation as an effective rhythm-control strategy for AF, particularly in patients with symptomatic paroxysmal AF refractory to antiarrhythmic drug therapy. Early landmark trials demonstrated that RFA targeting the pulmonary veins was superior to pharmacological therapy for maintaining sinus rhythm and improving quality of life [57,58]. These studies provided the first randomized evidence that catheter-based PVI, rather than focal arrhythmia suppression, was the critical mechanistic target in AF ablation.

Subsequent multicenter trials refined RFA techniques and broadened indications. Studies such as those led by Martin Karch and colleagues demonstrated that wide antral circumferential RFA showed no superiority in safety and efficacy compared with segmental approaches [59], while randomized comparisons confirmed the superiority of RFA over continued antiarrhythmic drug therapy for freedom from recurrent AF [3]. Importantly, these trials also clarified that success rates were higher in paroxysmal than in persistent AF [59], emphasizing the role of atrial substrate progression in long-term outcomes.

Later randomized trials focused on technology optimization, including irrigated RF catheters, contact-force sensing, and lesion-quality metrics. Studies evaluating contact-force guided RFA showed improved procedural consistency and reduced acute pulmonary vein reconnection compared with conventional RF techniques [60]. Parallel trials comparing RFA with CBA demonstrated broadly similar efficacy and safety, reinforcing RFA as a benchmark against which alternative energy sources are measured [14].

More recent randomized trials extended RFA into earlier disease stages. Trials enrolling treatment-naïve patients demonstrated that first-line RFA was superior to antiarrhythmic drugs for preventing AF recurrence and reducing arrhythmia burden [4]. In parallel, outcome-driven studies such as CASTLE-AF showed that RFA in patients with AF and heart failure reduced mortality and heart-failure hospitalization, marking a shift from symptom control toward prognostic benefit in selected populations [5]. Collectively, these clinical trials established RFA as an effective, durable, and increasingly disease-modifying therapy for AF, forming the clinical foundation upon which newer ablation technologies have been developed and compared.

### 4.2. Cryoablation: Clinical Studies

Randomized clinical trials have established cryothermal ablation as an effective and safe alternative to RFA for the treatment of AF, particularly in patients with paroxysmal AF. Early clinical development focused on the unique biophysical properties of cryoenergy, notably tissue adherence during freezing and homogeneous lesion formation, which facilitated reproducible PVI. Initial randomized trials comparing cryothermal ablation with antiarrhythmic drug therapy demonstrated superior freedom from recurrent AF and improved symptom control with catheter ablation, supporting cryoablation as a viable rhythm-control strategy [61].

Subsequent multicenter randomized trials evaluated CBA specifically, standardizing PVI as a single-shot approach. Trials such as STOP AF and STOP AF First demonstrated that cryoballoon PVI was superior to antiarrhythmic drug therapy in maintaining sinus rhythm, both as second-line and first-line therapy, with acceptable complication rates [61,62]. These studies showed that durable electrical isolation could be achieved with relatively short procedure times and reduced operator dependence compared with point-by-point RFA.

Head-to-head randomized comparisons between CBA and RFA further clarified the role of cryotherapy. The landmark FIRE AND ICE trial demonstrated non-inferiority of CBA compared with RFA for efficacy, with similar rates of freedom from atrial arrhythmias and major complications [14]. Importantly, CBA was associated with fewer repeat ablations and rehospitalizations, supporting its procedural durability. These findings established cryoablation as a benchmark technology alongside RF rather than a niche alternative.

More recent trials extended cryoablation into earlier disease stages and broader populations. First-line ablation trials showed that CBA was superior to antiarrhythmic drugs in preventing AF recurrence and reducing arrhythmia burden in treatment-naïve patients [16,62]. Safety analyses across trials consistently identified phrenic nerve palsy as a cryo-specific complication, but with low rates of permanent injury when contemporary monitoring strategies were applied [63]. Collectively, randomized clinical trials positioned cryoablation as an effective, reproducible, and guideline-endorsed approach to AF ablation, providing a critical comparator for newer non-thermal technologies such as PFA.

### 4.3. Pulsed Field Ablation: Clinical Studies

Clinical evaluation of PFA for AF has progressed rapidly from first-in-human feasibility studies to large randomized trials, driven by its fundamentally non-thermal mechanism and favorable pre-clinical safety profile. Early prospective human studies demonstrated that PFA could achieve acute PVI with high procedural success and minimal collateral injury, confirming the translational relevance of animal electroporation data [64]. These initial trials established feasibility, reproducibility, and a low incidence of esophageal or phrenic nerve injury, even with posterior left atrial applications.

Subsequent multicenter trials expanded the clinical evidence base. The PULSED AF trial evaluated PFA in patients with both paroxysmal and persistent AF, demonstrating high rates of acute PVI and acceptable arrhythmia-free survival at one year, with a notably low rate of serious adverse events [65]. Importantly, these studies reported an absence of atrio-esophageal fistula, pulmonary vein stenosis, or permanent phrenic nerve injury, reinforcing the tissue selectivity suggested by pre-clinical work. Parallel early commercial and investigational studies confirmed procedural efficiency, with short procedure times and limited need for extensive lesion titration.

The pivotal ADVENT trial [26] marked a key milestone by directly comparing PFA with conventional thermal ablation (RFA or CBA) in a randomized design. ADVENT demonstrated that PFA was non-inferior to thermal ablation for freedom from atrial arrhythmias at 12 months, while meeting prespecified safety endpoints. Although overall major complication rates were similar between groups, PFA showed fewer thermal injury-related events, supporting its differentiated safety profile [26]. These findings positioned PFA not merely as an experimental technology but as a viable alternative to established ablation modalities.

More recent randomized and registry-supported studies have continued to evaluate PFA across broader AF populations and catheter designs, with consistent findings of comparable efficacy, high procedural reproducibility, and favorable safety [66,67]. Collectively, these clinical trials establish PFA as an effective rhythm-control strategy for AF and provide the clinical foundation for its comparison with RFA and cryothermal ablation in contemporary practice.

## 5. Clinical Outcomes: Comparative Evidence and Meta-Analyses

### 5.1. Observational Comparisons

The study by Maurhofer and colleagues [29] performed a propensity score-matched comparison of patients with paroxysmal AF who underwent first-ever PVI using one of three energy modalities: PFA, CBA, or RFA. The goal was to evaluate procedural characteristics, safety metrics, and one-year clinical outcomes in a real-world setting.

The authors included 200 patients in total: 40 treated with PFA, 80 with CBA, and 80 with RFA, matched on clinical variables to minimize baseline differences between groups. Standardized ablation protocols were used: a 32-application lesion set for PFA, a time-to-effect plus 2 min strategy for CBA, and the CLOSE protocol for RFA. Follow-up consisted of scheduled 7-day Holter ECG monitoring at 3, 6, and 12 months post-procedure to detect atrial tachyarrhythmia recurrence.

In terms of procedural efficiency, the study found clear differences between techniques. CBA had the shortest median procedure time (75 min), followed by PFA (94 min), with RFA taking the longest (182 min) (*p* < 0.001). Fluoroscopy exposure also differed across groups: RFA had the lowest radiation dose, with higher doses seen in the PFA and CBA groups. These findings mirror prior evidence that single-shot modalities can reduce procedure duration compared with point-by-point RF delivery.

Regarding one-year outcomes, freedom from atrial tachyarrhythmia was 85.0% in the PFA group, 66.2% in the CBA group, and 73.8% in the RFA group. Although these differences did not reach statistical significance when comparing PFA with CBA (*p* = 0.12) or RFA (*p* = 0.27), the numerical trend favored PFA at 12 months. Overall, the analysis suggests that PFA achieves at least comparable arrhythmia-free survival at one year relative to established thermal ablation strategies in patients with paroxysmal AF.

Safety profiles were acceptable across all modalities, with no indication of higher complication rates associated with PFA. While the study was not powered to detect small differences in rare adverse events, the observed safety data add to the growing body of evidence supporting the favorable procedural risk profile of PFA seen in other registries and early comparative analyses.

In summary, the propensity-matched comparison demonstrates that PFA for PVI yields comparable one-year efficacy to CBA and RFA in patients with paroxysmal AF while offering procedural advantages in terms of efficiency (Table 2). These results add to the evidence base suggesting that the newer, non-thermal PFA modality may be an effective alternative to conventional thermal ablation in this population, with similar mid-term arrhythmia-free survival and acceptable safety outcomes.

### 5.2. Head-to-Head Randomized Trials

The **ADVENT Trial** [26] was a multicenter, randomized, controlled, non-inferiority study designed to compare PFA with conventional thermal ablation for pulmonary vein isolation in patients with paroxysmal atrial fibrillation undergoing a first catheter ablation procedure. The trial was motivated by the emergence of PFA as a non-thermal ablation modality with the potential to maintain efficacy while reducing collateral tissue injury associated with RFA and CBA.

Patients with symptomatic paroxysmal atrial fibrillation refractory to at least one antiarrhythmic drug were randomized in a 1:1 fashion to undergo pulmonary vein isolation using either a PFA system or standard-of-care thermal ablation. The thermal ablation arm allowed operator-selected use of RFA or CBA, reflecting contemporary real-world practice. All procedures were performed according to standardized protocols, and pulmonary vein isolation was the mandatory lesion set in both groups.

The trial had dual primary endpoints, assessing both effectiveness and safety. The effectiveness endpoint was freedom from documented atrial tachyarrhythmia recurrence after a standard blanking period, without the need for repeat ablation or escalation of antiarrhythmic drug therapy. The safety endpoint was a composite of serious procedure- or device-related adverse events, including death, stroke or transient ischemic attack, cardiac tamponade, pulmonary vein stenosis, atrio-esophageal fistula, or other major complications. The study was powered to test the non-inferiority of PFA relative to thermal ablation for both endpoints.

At 12 months of follow-up, PFA met the prespecified criteria for non-inferiority to thermal ablation with respect to arrhythmia-free survival. Rates of freedom from atrial tachyarrhythmia were comparable between groups, demonstrating that myocardial lesion formation achieved with electroporation was clinically as effective as that produced by thermal injury. Importantly, procedural success in achieving acute pulmonary vein isolation was high and similar across both treatment arms.

With respect to safety, PFA was also non-inferior to thermal ablation for the composite serious adverse event endpoint. Notably, certain complications traditionally associated with thermal energy—such as pulmonary vein narrowing and esophageal injury—were numerically lower in the PFA group, consistent with the non-thermal, tissue-selective mechanism of electroporation. Although the trial was not powered to detect differences in rare individual complications, the overall safety profile of PFA was favorable and aligned with preclinical expectations.

Procedural characteristics differed between groups. PFA was associated with shorter procedure times compared with thermal ablation, reflecting the rapid lesion delivery and reduced need for point-by-point energy application. Fluoroscopy time and radiation dose varied by operator and technique but were generally acceptable in both arms. These workflow advantages suggest potential efficiency benefits of PFA in routine clinical practice.

In conclusion, the ADVENT Trial demonstrated that PFA is as effective and as safe as conventional thermal ablation for PVI in patients with paroxysmal AF (Table 2). By achieving non-inferior arrhythmia control with a comparable or potentially improved safety profile and greater procedural efficiency, the trial established PFA as a viable alternative to RFA and CBA. These results represent a pivotal step toward broader adoption of non-thermal ablation strategies in AF management, while highlighting the need for longer-term follow-up to assess lesion durability and late clinical outcomes.

The **SINGLE SHOT CHAMPION Trial** [68] was a prospective, randomized, multicenter clinical study designed to directly compare PFA with CBA for PVI in patients with paroxysmal AF undergoing a first catheter ablation. The rationale for the trial was to evaluate whether a novel, non-thermal, single-shot ablation technology could achieve efficacy and safety outcomes comparable to those of an established single-shot thermal approach, while potentially offering procedural advantages related to tissue selectivity and workflow efficiency.

Eligible patients had symptomatic paroxysmal AF refractory to antiarrhythmic drug therapy and no prior left atrial ablation. Participants were randomized in a 1:1 fashion to undergo PVI using either a multielectrode PFA catheter delivering pulsed electric fields or a second-generation cryoballoon catheter using cryothermal energy. PVI was the sole mandatory lesion set in both arms, reflecting a standardized and clinically relevant ablation strategy for paroxysmal AF.

The primary efficacy endpoint was freedom from documented atrial tachyarrhythmia recurrence after a conventional blanking period, assessed during follow-up using rhythm monitoring and clinical evaluation. The trial was designed to test non-inferiority of PFA compared with CBA. Secondary endpoints included acute procedural success, procedural metrics such as procedure duration and fluoroscopy exposure, and safety outcomes.

At one year of follow-up, PFA met the prespecified criteria for non-inferiority compared with CBA with respect to arrhythmia-free survival. Rates of freedom from atrial tachyarrhythmia were similar between the two groups, demonstrating that durable pulmonary vein isolation could be achieved with both electrical and cryothermal single-shot technologies. Acute isolation of all targeted pulmonary veins was high and comparable in both treatment arms.

Procedural characteristics differed in ways consistent with the underlying technologies. PFA was associated with shorter overall procedure times, reflecting the rapid delivery of energy and the absence of prolonged freezing cycles required with CBA. Fluoroscopy time and radiation exposure were acceptable in both groups, although operator experience and workflow differences influenced these metrics.

With respect to safety, both ablation strategies demonstrated favorable profiles with low rates of serious adverse events. No excess of major complications was observed in the PFA group. Importantly, complications classically associated with thermal injury—such as phrenic nerve palsy or esophageal damage—were infrequent, and the overall findings were consistent with the hypothesized tissue-selective nature of PFA. As with other contemporary ablation trials, the study was not powered to detect small differences in rare adverse events.

In summary, the SINGLE SHOT CHAMPION Trial showed that PFA is non-inferior to CBA for PVI in patients with paroxysmal AF. By combining comparable one-year efficacy with a favorable safety profile and shorter procedure times, the trial established PFA as a credible alternative to CBA within a single-shot PVI paradigm (Table 2). These results complement findings from broader comparative studies and support the expanding role of non-thermal ablation technologies in the treatment of AF, while underscoring the importance of longer-term follow-up to assess durability and late outcomes.

The **BEAT PAROX-AF Trial** [66] is a European multicenter, prospective, superiority, open-label randomized clinical study designed to compare the efficacy and safety of PFA versus optimized point-by-point RFA for PVI in patients with drug-resistant, symptomatic paroxysmal AF. The trial enrolled approximately 292 patients across high-volume centers, randomizing them to receive either a single-shot PFA using a pulsed-field electroporation system or conventional RFA with an advanced contact-force sensing catheter and the CLOSE protocol for RFA lesion creation, reflecting state-of-the-art thermal ablation practice.

The primary effectiveness endpoint was single-procedure success at 12 months, defined as freedom from documented atrial tachyarrhythmia recurrence after a standard post-ablation blanking period without the need for repeat ablation or escalation of antiarrhythmic therapy. Secondary endpoints included procedural characteristics (procedure time, fluoroscopy exposure) and safety measures (serious adverse events including tamponade, stroke, phrenic nerve injury, or other major complications).

At one-year follow-up, the trial demonstrated that PFA and optimized RFA produced nearly identical efficacy, with 77.2% single-procedure success in the PFA arm versus 77.6% in the RFA arm (adjusted difference approximately 0.9%, 95% CI −8.2% to 10.1%, *p* = 0.84), indicating no statistically significant superiority or inferiority in arrhythmia-free survival. This result indicates that PFA, despite its novel non-thermal electroporation mechanism, achieved comparable clinical outcomes to one of the most refined contemporary RFA protocols in patients with paroxysmal AF.

In terms of procedural performance, PFA was associated with shorter overall procedure times compared with RFA (for example, average PFA ~56 min vs. RFA ~95 min in reported interim analyses), reflecting the rapid, single-shot nature of pulsed-field energy delivery. Fluoroscopy exposure was somewhat higher with PFA in some centers, likely due to differences in mapping and catheter manipulation workflows between technologies.

With respect to safety, the overall rate of serious adverse events was lower numerically in the PFA group (e.g., 3.4% vs. 7.6% with RFA), though the study was not powered for formal comparisons of rare complications. Notably, the pattern of complications aligned with expectations for each technology, with PFA’s non-thermal mechanism theoretically reducing risks of collateral thermal injury while RFA’s long history of use presented the expected safety profile.

In summary, the BEAT PAROX-AF Trial provides the first large randomized head-to-head comparison of PFA versus optimized RFA in paroxysmal AF, showing comparable one-year efficacy and acceptable safety profiles between the two energy sources (Table 2). The findings support the clinical viability of PFA as an alternative to intricate point-by-point RFA strategies in this patient population, with the added procedural efficiency inherent to non-thermal single-shot lesion delivery. 

### 5.3. Meta-Analyses of Randomized Controlled Trials

Two contemporary meta-analyses have specifically pooled randomized controlled trial data to compare PFA with conventional thermal ablation. The analysis by Patrick Badertscher et al. [27] focused on paroxysmal AF and combined the available RCTs comparing PFA with thermal ablation (predominantly RFA, with some inclusion of cryoablation). The primary efficacy endpoint—freedom from atrial arrhythmia at approximately 12 months—demonstrated non-inferiority of PFA compared with thermal modalities. Importantly, no statistically significant difference was observed in major adverse cardiac or cerebrovascular events, indicating comparable procedural safety. Procedural efficiency favored PFA, with shorter overall procedure times.

The meta-analysis by Rasha Kaddoura et al. [28] broadened the comparison to PFA versus both RFA and CBA, again restricting inclusion to randomized trials. Efficacy outcomes were consistent with Badertscher et al. [27], showing no significant difference in arrhythmia recurrence or arrhythmia-free survival between PFA and either thermal strategy. Safety analyses revealed similar overall rates of major complications, but with a notable qualitative pattern: PFA showed fewer collateral-tissue injury events, particularly esophageal injury and pulmonary vein stenosis, although these outcomes were infrequent and underpowered for definitive statistical separation.

Across both RCT-only meta-analyses, the central message is consistent. PFA achieves rhythm-control efficacy equivalent to RFA and CBA, without an increase in serious adverse events. While large differences in hard safety endpoints were not demonstrated, signals favoring improved tissue selectivity with PFA were reproducible (Table 3). However, both analyses emphasize that conclusions are constrained by the small number of available RCTs and limited long-term follow-up, underscoring the need for additional randomized evidence before definitive superiority claims can be made.

## 6. Conclusions

Radiofrequency ablation and cryoablation have established catheter ablation as an effective and durable therapy for atrial fibrillation, supported by extensive mechanistic validation, randomized clinical trials, and long-term clinical experience. Both modalities reliably achieve pulmonary vein isolation but remain constrained by the non-selective nature of thermal injury, which underlies residual risks of collateral tissue damage despite ongoing technological refinement.

Pulsed field ablation introduces a fundamentally different approach based on irreversible electroporation, enabling rapid, myocardial-selective lesion formation with relative preservation of surrounding structures. Preclinical studies provide a strong mechanistic rationale for this tissue selectivity, and contemporary randomized trials demonstrate clinical efficacy comparable to radiofrequency and cryoablation, along with procedural efficiency and a favorable safety profile. Recent head-to-head trials and meta-analyses of randomized controlled studies reinforce the consistency of these findings.

While early and mid-term outcomes support pulsed field ablation as a viable alternative to thermal ablation technologies, important questions remain regarding long-term lesion durability, performance in persistent atrial fibrillation, and broader real-world applicability. As clinical experience expands and longer-term follow-up data become available, the relative positioning of pulsed field ablation alongside established thermal modalities will continue to evolve. A nuanced understanding of the biophysical and clinical trade-offs among these technologies will be critical for optimizing patient selection and advancing atrial fibrillation ablation strategies.

## Figures and Tables

**Table 1:** Comparative characteristics of radiofrequency ablation, cryoablation and pulsed field ablation.

Feature	RF Ablation	Cryoablation	Pulsed Field Ablation
Energy type	Thermal (heat)	Thermal (cold)	Electrical (non-thermal)
Primary injury	Protein denaturation	Ice crystals + ischemia	Membrane electroporation
Cell death	Coagulative necrosis	Necrosis + apoptosis	Apoptosis/necrosis
ECM preservation	No	Yes	Yes
Tissue selectivity	No	No	Yes (myocyte predominant)
Lesion speed	Seconds–minutes	Minutes	Seconds
Lesion Characteristics	Non-homogenous, with extensive fibrosis	More homogeneous, well-demarcated	Homogenous, contiguous, low fibrosis
Collateral injury risk	Higher	Moderate	Lowest (current data)

ECM—extracellular matrix; RF—radiofrequency.

**Table 2:** Propensity score-matched studies and randomized controlled trials comparing the effectiveness and safety of pulsed field ablation with thermal ablation modalities.

Study	Comparators	Primary Efficacy Endpoint	Efficacy Result	Major Safety Endpoint(s)	Safety Result
Maurhofer et al. 2024 [29] (Observational, propensity score-matched study)	PFA vs. RFA vs. CBA	Freedom from atrial tachyarrhythmia at 12 months	No difference for PFA vs. thermal; Numerical trend favors PFA.	Pericardial tamponade	2 cases in PFA group
ADVENT Trial, 2023 [26] (RCT)	PFA vs. RFA and CBA	Freedom from arial tachyarrhythmia recurrence at 12 months	PFA non-inferior to thermal ablation	Composite of serious procedure or device-related adverse events (death, stroke or transient ischemic attack, cardiac tamponade, pulmonary vein stenosis, atrio-esophageal fistula, or other major complications)	PFA non-inferior to thermal ablation; numerically fewer esophageal/PV injuries with PFA; 1 death in PFA group.
SINGLE SHOT CHAMPION Trial, 2025 [68] (RCT)	PFA vs. CBA	Freedom from atrial tachyarrhythmia recurrence at 12 months assessed with rhythm monitoring	PFA non-inferior to CBA	Composite safety endpoint at 30 days (cardiac tamponade requiring intervention, persistent phrenic nerve injury, major vascular complications, stroke or transient ischemic attack, atria-esophageal fistula, or death)	Low rates of serious adverse events; no excess of major complications with PFA
BEAT PAROX-AF Trial, 2026 [66] (RCT)	PFA vs. RFA	Single-procedure success at 12 months (freedom from documented atrial tachyarrhythmia without the need for repeat ablation or escalation of antiarrhythmic therapy)	PFA demonstrated comparable efficacy to RFA (criteria for PFA superiority were not met)	Serious adverse events (tamponade, stroke, phrenic nerve injury, or other major complications)	No statistically significant difference; serious adverse events numerically lower in the PFA group (3.4% with PFA vs. 7.6% with RFA)

CBA—cryoballoon ablation, PFA—pulsed field ablation, and RFA—radiofrequency ablation.

**Table 3:** RCT-only meta-analyses for PFA effectiveness and safety.

Meta-Analysis	Comparators	Primary Efficacy Endpoint	Efficacy Result	Major Safety Endpoint(s)	Safety Result
Badertscher et al. 2025 [27]	PFA vs. thermal ablation (RFA ± CBA)	Freedom from atrial arrhythmia at 12 months	Non-inferior PFA vs. thermal	Composite serious adverse events (tamponade, stroke, PV stenosis, death)	No significant difference
Kaddoura et al. 2025 [28]	PFA vs. RFA and CBA	Arrhythmia recurrence/freedom from AF	Comparable efficacy across modalities	Major complications; procedure-related injury	Comparable overall safety; numerically fewer esophageal/PV injuries with PFA

AF—atrial fibrillation, CBA—cryoballoon ablation, PFA—pulsed field ablation, PV—pulmonary veins, and RFA—radiofrequency ablation.

## Data Availability

No new data were created or analyzed in this study. Data sharing is not applicable.

## Abbreviations

The following abbreviations are used in this manuscript:

AF	Atrial fibrillation
CBA	Cryoballoon ablation
Cryo	Cryoablation
ECM	Extracellular matrix
N	Nitrogen
PFA	Pulsed field ablation
PV	Pulmonary veins
PVI	Pulmonary vein isolation
RF	Radiofrequency
RFA	Radiofrequency ablation
Temp	Temperature
Vol	Volume
